# Complying with ISO 26262 and ISO/SAE 21434: A Safety and Security Co-Analysis Method for Intelligent Connected Vehicle

**DOI:** 10.3390/s24061848

**Published:** 2024-03-13

**Authors:** Yufeng Li, Wenqi Liu, Qi Liu, Xiangyu Zheng, Ke Sun, Chengjian Huang

**Affiliations:** 1 School of Computer Engineering and Science, Shanghai University, Shanghai 200444, China; liyufeng_shu@shu.edu.cn (Y.L.); liuwenqi@shu.edu.cn (W.L.); liuq@shu.edu.cn (Q.L.); zhengxy@shu.edu.cn (X.Z.); chengjian_huang@shu.edu.cn (C.H.); 2 The Purple Mountain Laboratories, Nanjing 211111, China

**Keywords:** intelligent connected vehicle, system-theoretic process analysis, ISO 26262, ISO/SAE 21434, loss scenario tree

## Abstract

A cyber-physical system (CPS) integrates communication and automation technologies into the operational processes of physical systems. Nowadays, as a complex CPS, an intelligent connected vehicle (ICV) may be exposed to accidental functional failures and malicious attacks. Therefore, ensuring the ICV’s safety and security is crucial. Traditional safety/security analysis methods, such as failure mode and effect analysis and attack tree analysis, cannot provide a comprehensive analysis for the interactions between the system components of the ICV. In this work, we merge system-theoretic process analysis (STPA) with the concept phase of ISO 26262 and ISO/SAE 21434. We focus on the interactions between components while analyzing the safety and security of ICVs to reduce redundant efforts and inconsistencies in determining safety and security requirements. To conquer STPA’s abstraction in describing causal scenarios, we improved the physical component diagram of STPA-SafeSec by adding interface elements. In addition, we proposed the loss scenario tree to describe specific scenarios that lead to unsafe/unsecure control actions. After hazard/threat analysis, a unified risk assessment process is proposed to ensure consistency in assessment criteria and to streamline the process. A case study is implemented on the autonomous emergency braking system to demonstrate the validation of the proposed method.

## 1. Introduction

Nowadays, the intelligence and connectivity of vehicles have significantly enhanced the driving experience, but at the same time, they have also brought more cybersecurity issues. Indeed, for intelligent connected vehicles (ICVs), safety risks are no longer limited to accidental functional failures, and cyberattacks can also cause safety risks, resulting in physical injuries [1,2,3].

The exchange of information between vehicles and the outside world is called Vehicle-to-Everything (V2X), which includes various interaction modes such as Vehicle-to-Vehicle (V2V), Vehicle-to-Infrastructure (V2I), and Vehicle-to-Network (V2N). As V2X communication technology continues to evolve, vehicles have more and more interfaces with the external world, leading to more entry points for cyberattacks. The expansion of the attack surface leads to an increasing probability of attacks on in-vehicle systems as well [4]. According to the Global Automotive Cybersecurity Report [5] released by Upstream Security in 2023, the complexity of attacks targeting vehicles continues to rise, with 97% of these attacks being initiated remotely. This means that an attacker only needs to connect to the in-vehicle network to launch an attack. Researchers exploited multiple vulnerabilities in the in-vehicle infotainment and telematics box to remotely attack a BMW vehicle and gain root privileges [6].

In addition, ICVs are equipped with a number of driver assistance functions to perform specific driving tasks. These automated functions are controlled and managed by electronic control units (ECUs) interconnected through the controller area network (CAN) bus, which collects vehicle and environmental information from sensors for decision-making [7]. Modern vehicles are equipped with more than 100 ECUs, while the lines of code are increasing rapidly. According to research, the lines of code in vehicles will approach 700 million by 2025–2030, up from 100 million in 2015 [8]. The increasing complexity of systems makes it more difficult to design a security system with fewer vulnerabilities. Attackers can exploit vulnerabilities in the system to gain unauthorized access to the system and tamper with data, which can lead to accidents. Nie et al. experimentally demonstrated how to remotely control an ECU by sending arbitrary CAN packets to a Tesla vehicle updated with the latest firmware [9].

However, the safety and security of vehicles are typically handled by different teams, which can lead to varying degrees of conflict in the analysis results [10]. For example, complex encryption algorithms enhance data security but also increase latency. OTA update allows vehicle manufacturers to remotely update a vehicle’s software to fix bugs and provide new features, but it also provides an entry point for cyberattacks [11]. Therefore, safety and security (S&S) co-analysis is necessary, which allows analysts to identify and optimize solutions in a timely manner to achieve trade-off objectives. Existing co-analysis methods have often been adapted from safety or security analysis methods. AFT (attack fault tree) [12] combines safety properties from fault trees and security conditions from attack trees. SAHARA (security-aware hazard analysis and risk assessment) [13] combines HARA (hazard analysis and risk assessment) from the safety and the STRIDE approach from the security domain. However, these methods exhibit limitations when applied to complex systems, as they may overlook hazards stemming from unsafe/unsecure interactions between components. On the other hand, STPA-SafeSec [14] integrates STPA (system-theoretic process analysis) [15] and STPA-sec [16] into one concise framework, linking the abstract control structure to the physical system design to integrate the results from traditional security analysis methods. Although STPA-SafeSec cannot adequately emphasize the connectivity of ICVs when analyzing them, the STPA-based approach demonstrates advantages when analyzing complex systems. The main idea of STPA is to view safety problem as a dynamic control problem and focus on unsafe interactions between components.

In the traditional vehicle industry, functional safety engineering methods and processes have become an industry standard [17]. The functional safety standard for road vehicles, ISO 26262 [18], is designed to address functional failures in the electrical and electronic (E/E) systems of vehicles. ISO 26262 requires safety engineers to perform the HARA process during the concept phase, which focuses on identifying potential hazards and evaluating risks in E/E systems. ISO/SAE 21434 [19] is a vehicle cybersecurity standard designed to address cyberattacks against vehicles. ISO/SAE 21434 performs the threat analysis and risk assessment (TARA) process during the concept phase with the aim of identifying potential security threats and evaluating their risk levels. ISO 26262 and ISO/SAE 21434 are both important standards for the vehicle industry that focus on functional safety and cybersecurity, respectively. They provide specifications and guidance to vehicle manufacturers and suppliers to help them ensure that vehicle systems meet the necessary functional safety requirements and cybersecurity requirements during design, development, and operation. Typically, the HARA and TARA processes are handled by different teams, which can have some shortcomings:

1. Lack of information sharing [20]: If there is not enough information sharing between the two teams, it can lead to duplication of effort or conflicting information, which can affect the quality of the overall analysis process.
2. Differences in analysis methods [21]: There may be differences in the analytical methods used by the HARA and TARA teams. If there is no consensus between the two teams, this may make it difficult to harmonize the results of the analysis.
3. Lack of comprehensive analysis [22]: If the two teams do not conduct an effective integrated analysis, it may result in an inability to adequately consider the correlations and interactions between safety and security, which may affect the assessment and management of the overall risk to the system.

In addition to these shortcomings, some mandatory regulations emphasize the necessity of conducting safety and security co-analyses. In March 2021, the United Nations Economic Commission for Europe (UNECE) published the WP.29 regulations, two documents covering key future topics in the automotive domain: Cybersecurity R155 [23] and Software Update R156 [24]. UNECE R156 comes along with UNECE R155 to ensure that the manufacturer put in place appropriate safety and security processes for conducting software updates [25]. Furthermore, the UNECE R155 and UNECE R156 point to ISO/SAE 21434 as the generic cybersecurity risk management framework.

In summary, due to the practical needs of vehicle safety and mandatory regulations, there is an urgent need for a S&S co-analysis method that complies with international standards.

In this work, we proposed an S&S co-analysis method for ICVs. Our method is compliant with the HARA (ISO 26262) and TARA (ISO/SAE 21434) processes and helps to comprehensively identify and assess the loss scenarios of the system. It has made the following contributions:

1. We improved STPA by adding a physical component diagram to describe the component interactions of ICVs while highlighting the ICV’s connections to the external world. In addition, we proposed loss scenario trees for describing fault propagation paths and attack paths, which are used to obtain more detailed safety and security requirements.
2. Our approach combines HARA and TARA in the same process, which compensates for the lack of information sharing and analytical synthesis. A unified risk assessment process has been added to our method. The risk level of events at the root of the loss scenario tree is evaluated using a unified risk matrix and the results are mapped to the automotive safety integrity level (ASIL) of ISO 26262 and the security risk level (SecRL) of ISO/SAE 21434.
3. A case study of the autonomous emergency braking (AEB) system on our experimental vehicle platform shows that our method can effectively support the concept phase of the vehicle development process.

The rest of this work is structured as follows. In Section 2, we summarized previous works and compared them. Section 3 details the flow of our method and discusses how it complies with the concept phase analysis process in international standards. Section 4 demonstrates the application of our method to the AEB system. Finally, Section 5 is the conclusion and future work of our research.

## 2. Related Work

According to the focus of the analysis methods, we categorize the methods into three groups: safety analysis methods focusing on the functional failure of system components, security analysis methods focusing on malicious attacks, and S&S co-analysis methods. Table 1 lists comparative information on some of these methods. We provide a brief description of the methods and compare the nature of the methods (qualitative/quantitative), causal factors, level of analysis (abstract/specific), and whether they are aligned with the standards.

### 2.1. Safety Analysis Methods

Traditional safety analysis methods, such as failure mode and effect analysis (FMEA) [31], fault tree analysis (FTA) [32], and hazard and operability analysis (HAZOP) [33], are typically used to analyze the safety and reliability of a system. FMEA is a bottom-up hazard analysis method used to identify failure modes, effects, and causes of a system. The analysis begins with components to determine possible failure modes and consequences so that mitigation strategies can be developed in advance. FTA is a top–down analysis approach that turns system failures into combinations of hazardous events. HAZOP is a process hazard analysis technique that decomposes a system into different nodes and operational steps. It uses guide words to identify situations that may cause deviations from the expected process or operational state.

However, these methods are not entirely suitable for analyzing complex cyber-physical systems (CPSs). Applying HAZOP and FTA to software-intensive systems has proven to be error-prone [34]. In addition, FMEA and FTA are based on the failure chain, in which each hazard or accident is considered to be caused by component failures. However, for ICVs, unsafe interactions between components can also lead to hazards. In other words, reliability may not be consistent with safety. STPA [15] is a hazard analysis method based on the system-theoretic accident model and processes (STAMP), which focuses on emergent properties (properties that only appear when system components interact). In addition to component failures, STPA also assumes that accidents can result from unsafe interactions between system components, even if no component experiences a failure. There are four main steps in conducting an analysis using STPA: define purpose of the analysis, model the control structure, identifying unsafe control actions, and identifying loss scenarios.

Mahajan et al. [35] investigated the application of STPA in a Lane Keeping Assist (LKA) system to identify the design constraints and requirements needed to design safer systems. Abdulkhaleq and Wagner [36] applied STPA to the Adaptive Cruise Control (ACC) system of vehicles in order to obtain potential directions for improvement of the ACC system. Sharma et al. [37] introduced the STPA process for generating design requirements for the Automatic Emergency AEB system, which provides an improved structured approach for scenario analysis. To address the problem that STPA does not include the risk assessment required by international standards for vehicles, Chen et al. [26] proposed a new method called system-theoretic process analysis based on FMEA templates (STPAFT). A case study of the Fuel Level Estimation and Display System demonstrates that STPAFT can effectively support the concept phase of ISO 26262.

### 2.2. Security Analysis Methods

Security analysis methods do not consider the impact on safety when assessing risks of traditional IT infrastructure. However, malicious attacks against vehicles may have an impact on the functions of the vehicle and the personal safety. Attack Tree [38] is a widely used method for security analysis. Attack Tree uses the same basic idea as FTA to represent the steps of the attack process in the form of a tree. Karray et al. uses graph transformations to formally model the vehicle architecture and its state evolution in order to investigate cyber-physical attacks against it. The generated attacks are transformed into attack trees that are used to estimate the overall risk of the system [39].

In the field of vehicle security, Henniger et al. [40] proposed EVITA to assess the risk level of an attack through attack probability and impact. However, EVITA mainly focuses on security risk assessment and does not use a systematic approach for the identification of threat scenarios, so it may be more exhausting when identifying threat scenarios for ICVs. The risk analysis method for cooperative engines (RACE) [41] is an improved approach for EVITA. RACE defines the severity value as the highest of the four values of the severity vector. As an extension to EVITA, RACE also improves the EVITA attack tree to include a brief and practical description of the attack. Monteuuis et al. [42] proposed a systematic threat analysis and risk assessment framework called SARA. This framework includes improved threat models, new attack methods/asset graphs, attacker profiles, and a new metrics called observations. Finally, the feasibility of the method in safety analysis is validated by two cases: vehicle tracking and emergency braking failure. Cui et al. proposed a security risk analysis method called vehicles risk analysis (VeRA) to assess the security risk of ICVs [27]. The risk level is determined based on the probability, severity, and controllability corresponding to the attack. This work discusses the classification of human control under different human capabilities and vehicle automation levels. VeRA consumes less time and gives the same results as compared to EVITA. Sheik et al. [43] systematically evaluated TARA methods and applied the Spoofing, Tampering, Repudiation, Information disclosure, Denial of service, and Elevation of privileges (STRIDE) threat model and Damage, Reproducibility, Exploitability, Affected Users, and Discoverability (DREAD) risk assessment to cloud-assisted connected and autonomous vehicles (CCAVs). In addition, the research indicated that current analysis techniques often overlook the relationships between components in CCAVs.

One of the methods for assessing the security risk of a complex CPS is STPA-Sec [16]. Unlike traditional security analysis methods originating from the IT domain, STPA-Sec is based on systems theory. It is an extension of STPA, customized to include security analysis. STPA-Sec maintains the same four basic steps as STPA, but essentially introduces threat/vulnerability identification in the last step [44]. Sahay et al. [45] evaluated and compared the application of STPA-Sec, STRIDE, and CORAS in identifying threats and vulnerabilities to the cybership system. Based on the results of the analysis, STRIDE provides a structured approach to highlight threats to the system that can be combined with CORAS and STPA-Sec to make them more effective.

### 2.3. S & S Co-Analysis Methods

For a CPS, malicious attacks often exploit system vulnerabilities leading to catastrophic system functional failures. Therefore, it is critical to incorporate both safety and security into the system lifecycle. Li et al. [46] proposed an smart Dynamic Heterogeneous Redundancy (DHR) scheme that simultaneously improves the safety and security of ICV. Kumar and Stoelinga [12] proposed attack fault trees (AFTs), a form of combining fault trees and attack trees. The AFTs are also equipped with stochastic model verifying techniques, which enable extensive qualitative and quantitative analysis.

For the vehicle industry, a number of S&S co-analysis approaches have been proposed. SAHARA [13] combines the HARA process with the STRIDE threat model to track the impact of security issues on the concept of system-level safety. SAHARA is fully compliant with the process requirements of ISO 26262 while expanding the results of the analysis by taking security threats into account. ${\mathrm{US}}^{2}$ [28] is a unified approach to safety and security analysis. It uses the attack potential, threat criticality, and driving automation levels to assess the risk of an attack. Cui et al. proposed a collaborative analysis framework of safety and security, following ISO 26262 and SAE J3061 [47]. With this framework, it is possible to obtain a clear picture of the vehicle’s functions, structure, component failures, malicious attacks, as well as unaddressed vulnerabilities, which can help to improve in-vehicle safety and security from both research and engineering perspectives. Sabaliauskaite et al. [29] proposed an approach for integrated safety and security analysis, which is compliant with the international standards SAE J3016, SAE J3061, and ISO 26262. It integrates STPA into the concept phase of ISO 26262 while using a six-step model to maintain consistency between safety and security artifacts.

Friedberg et al. [14] proposed a new methodology called STPA-SafeSec for safety and security analysis of CPS. It integrates STPA and STPA-sec into a concise framework that allows for the detection of a wider range of loss scenarios. In addition, STPA-SafeSec overcomes the limitations of STPA by introducing security constraints in the analysis and mapping the control layer to the component layer. De Souza et al. [48] extended STPA using the STRIDE threat model to identify previously undetectable security loss scenarios and security requirements. Triginer et al. [30] presented the integration of the safety and cybersecurity analysis method by combining systems theory methods STPA and STPA-sec with the HARA and TARA (SAE J3061 [49]) reliability theory methods. Cui et al. [50] proposed an approach for aligning safety and security at early development phases considering the levels of driving automation. They used the Failure, Attack, and Countermeasure (FACT) graph to connect safety failures, security attacks, and the associated countermeasures.

Within the existing analysis methods, some of them miss certain hazards/threats and some of them require significant effort [51]. In addition, there is no detailed work on how to use STPA in a process that is compatible with ISO 26262 and ISO/SAE 21434. To address the aforementioned concerns, we have introduced a safety and security co-analysis method based on STPA.

## 3. The Proposed Method

### 3.1. Overview of the Method

This approach incorporates a risk assessment step following the hazard/threat analysis, ensuring its compliance with the requirements of ISO 26262 and ISO/SAE 21434 during the concept phase.

**Figure 1:**
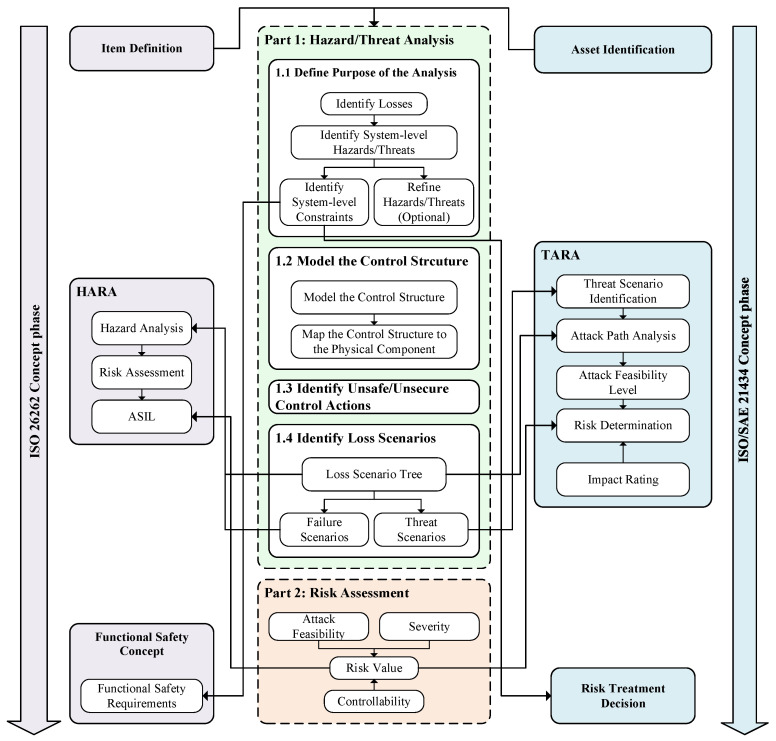
Flow diagram of the proposed method.

The flow diagram of the method is shown in Figure 1. To compensate for the lack of traditional STPA in risk assessment, our method is divided into two parts, with the first part involving an enhanced STPA process for hazard/threat analysis, and the second part focusing on the risk assessment of the analysis results. The artifacts that are produced (as output) and used (as input) by the steps are as follows:

1. Step 1: Hazard/Threat Analysis (enhanced STPA process)
  (a) Step 1.1: Define purpose of the analysis: At the beginning of the analysis process, the scope of the target system needs to be defined first. This can be obtained from item definition of HARA (from ISO 26262) and asset identification of TARA (from ISO/SAE 21434). With this step, system-level constraints can be initially obtained. This can provide the basis for the generation of functional safety requirements (required by ISO 26262) and cybersecurity requirements (required by ISO/SAE 21434).
  (b) Step 1.2: Model the control structure: Establish the control structure of the target system. The physical component diagram of the target system is obtained through the mapping from the control structure to the physical component diagram.
  (c) Step 1.3: Identify Unsafe/Unsecure Control Actions: Based on the control structure of the target system, the possible unsafe/unsecure control actions are analyzed and obtained.
  (d) Step 1.4: Identify loss scenarios: Based on the physical component diagram of the system, the possible loss scenarios are derived from the Loss Scenario Tree. This step corresponds to HARA’s hazard analysis and TARA’s threat scenario identification (threat scenarios are the reasons why threats arise, and some threats may have the same consequences as hazards) and attack path analysis.
2. Step 2: Risk assessment: For the identified loss scenarios, their risk values are obtained through a unified risk matrix and mapped to Automotive Safety Integration Level (ASIL, defined in ISO 26262) and Security Risk Level (SecRL, defined in ISO/SAE 21434), respectively.

The detailed explanation of the proposed method is provided in Section 3.3 and Section 3.4.

### 3.2. Foundations of ISO 26262, ISO/SAE 21434 and the Proposed Method

In Section 1, we mentioned that HARA and TARA often use different analysis methods, which may lack process interactions and result in poorly harmonized results. Our approach is a common one for both HARA and TARA. As shown in Table 2, our approach is based on the enhanced STPA, which shares a foundational similarity with the HARA and TARA processes. ISO 26262 employs HARA during the concept phase to identify potential functional failures in E/E systems that may result in hazards and assess their risk level. In contrast, ISO/SAE 21434 uses TARA to identify potential threats to vehicles and evaluate their risk levels, thereby establishing security requirements. They aim to integrate safety (ISO 26262) or security (ISO/SAE 21434) requirements in the early stages of system development (i.e., concept phase) to improve the safety or security of the system architecture and avoid costly rework when design flaws are discovered later. Similarly, our method can also be initiated at the concept phase to define safety and security requirements and constraints. And as the vehicle design is refined and more detailed decisions are made, the safety and security constraints can be gradually refined, and ultimately complete traceability from the requirements to all system artifacts can be easily maintained, thus enhancing the maintainability of the system. Therefore, it is plausible to infer that the proposed method could be highly compatible with the ISO 26262 and ISO/SAE 21434 concept phase analysis procedures.

### 3.3. Hazard/Threat Analysis

The four parts of this section correspond to the four steps of STPA and detail the hazard/threat analysis process of our method.

#### 3.3.1. Defining the Purpose of the Analysis

The first step in the whole process is aimed at defining the goals of the analysis, which mainly consists of identifying losses, identifying system-level hazards, identifying system-level safety/security constraints, and refining the constraints. Losses are crucial for identifying potential hazards/threats in the system, so that measures can be taken to prevent and minimize these losses. Losses can encompass various undesirable consequences, such as loss of human life, property damage, mission failure, and other consequences that stakeholders find unacceptable. Based on the identified losses, the associated hazards/threats can be identified. Hazards are usually caused by failures or malfunctions of system components, while threats are usually malicious attacks on the system. System-level hazards/threats will cause losses in specific environmental states and we limit them with safety/security constraints.

#### 3.3.2. Modeling the Control Structure

**Figure 2:**
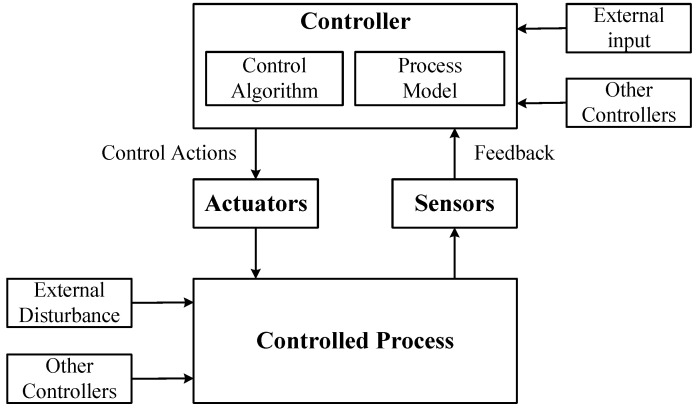
Generic control structure diagram.

The next step is to model the control structure for the target system, which is an abstract system model consisting of feedback control loops. The control structure mainly consists of the controller, actuators, controlled processes, and sensors, which are used to explain complex system component interactions. The generic control structure diagram is shown in Figure 2. A controller can provide control actions to control some processes. At the same time, a controller can also receive external inputs and can be controlled by other controllers. The control algorithm represents the controller’s decision-making process. The process model represents the internal beliefs used by the controller to make decisions.

Since the control structure is an abstract representation of the system, it focuses on the functional interaction and information feedback of the system, but ignores the details of the implementation. This may lead to insufficient understanding of the details of specific components, interfaces, and interactions.

**Figure 3:**
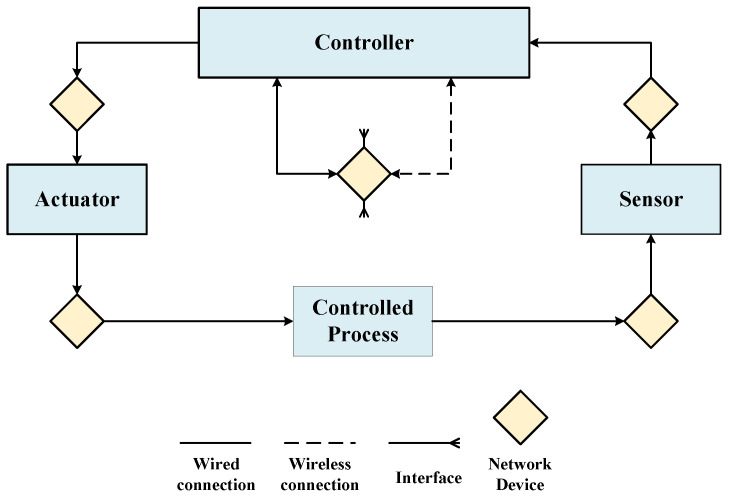
Generic physical component diagram.

In order to describe the system implementation and facilitate the analysis process, we proposed a physical component diagram for ICVs. After modeling the control structure diagram of traditional STPA, we map it to the system physical component diagram, which includes sensors, controllers, network devices, and connections related to system functions. Physical component diagrams describe real hardware and software, bringing hazard/threat analysis closer to the actual situation. At the same time, using physical component diagrams makes it easier to trace failure propagation paths and malicious attack paths, thus providing more comprehensive analysis and risk assessment. A generic physical component diagram is shown in Figure 3.

#### 3.3.3. Identifying Unsafe/Unsecure Control Actions

With the physical component diagram, we can analyze the control actions issued by the controller and identify the possible ways of unsafe/unsecure. Unsafe/Unsecure control actions (UCAs) can usually be traced to one or more hazards/threats. There are four ways a control action can be unsafe/unsecure:

1. Not Providing;
2. Providing;
3. Providing too early, too late, or in the wrong order;
4. Lasting too long or stopping too early.

#### 3.3.4. Identifying Loss Scenarios

After identifying the UCA, the next step is to determine the reason for its occurrence, which is usually due to (1) unsafe/unsecure controller behavior (e.g., controller failures, inadequate control algorithm, unsafe control input, and inadequate process model) and (2) inadequate feedback and other inputs (e.g., feedback or information not received and inadequate feedback is received). The identified loss scenarios generally include two categories, hazard scenarios focusing on accidental failure of system components and threat scenarios focusing on malicious attacks on the system.

Although STPA is able to effectively address the software-intensive characteristics of CPSs, it still has limitations. Specific unsafe/unsecure control actions are identified during the operation of the system, but STPA does not explain how malicious attacks or failures propagate and thus cause these unsafe/unsecure control actions. Control structures cannot describe how malicious attacks or failures propagate through complex systems. In addition, there may be some misunderstandings due to knowledge gaps and limited experience of safety/security analysts.

To address these shortcomings, we proposed a tree structure called loss scenario tree for identifying various scenarios that lead to UCAs. Describing the logical relationships between events in a tree structure helps to trace the chain of events in a systematic way.

**Figure 4:**
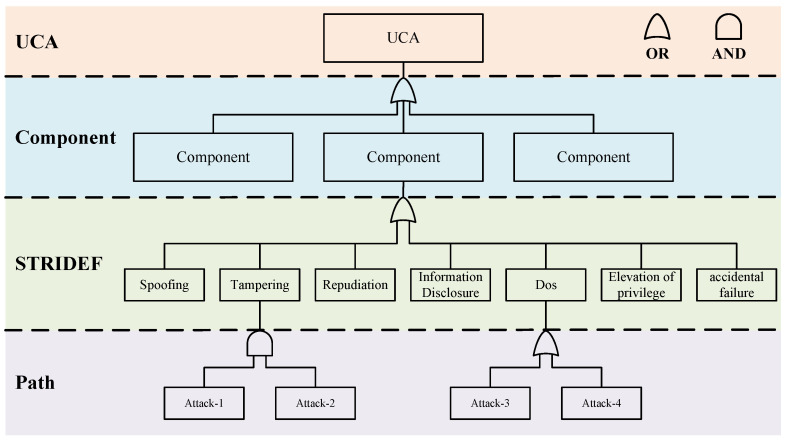
Loss scenario tree.

The loss scenario tree consists of four layers, the UCA layer, Component layer, STRIDEF (STRIDE+Failure) layer, and Path layer, as shown in Figure 4. Each layer of the structure is represented as follows:

1. UCA layer: The UCA as the root of the scenario tree is generated by the previous steps. We construct the entire loss scenario tree by analyzing the causes of UCA.
2. Component layer: At the component layer, we focus on critical components that may experience accidental failures or malicious attacks. It is important to note that the malfunction of a component or a combination of certain components can lead to UCA. In addition, a component may also consist of other subcomponents, which can be represented as several subtrees of this component.
3. STRIDEF layer: For components identified at the component level, if a malfunction occurs it may be due to a malicious attack or an accidental failure. At the STRIDEF (STRIDE+Failure) layer, we categorize threats into six well-defined categories based on the STRIDE (proposed by Microsoft) threat model, namely spoofing, tampering, repudiation, information leakage, denial of service, and elevation of privilege. This categorization helps us to have a clear picture of potential threats so that we can take appropriate security countermeasures. At this level, we also consider accidental component function failures (usually triggered by hardware aging, design flaws, etc., including random hardware failures and systematic failures in ISO26262), and this comprehensive analysis ensures the overall safety of the system.
4. Path layer: At the Path layer, we describe how a malicious attack or accidental failure occurs. We describe in detail the possible attack paths that an attacker could take and the fault propagation paths of the components. We can represent the relationship between each of the basic events more explicitly by using some logic gates (OR/AND). In the traditional component-based analysis, it is difficult to represent the interaction between components. Our method makes some improvements to address this issue. For some complex systems, if there are multiple components interacting with each other, we can still add the Component layer of a low-level component to the Path layer of a high-level component or reuse other existing analysis results.

Each layer of the loss scenario tree has a clear role. The UCA layer contains the things we do not want to happen. The Component layer contains the related components that may cause UCA. The STRIDEF layer categorizes the potential threats. The Path layer shows the logical relationship between the sub-events of a threat scenario through a tree structure, and we can also further describe the interactions between components at this layer. By considering multiple layers in depth, we can better understand the possible risks to the system so that we can develop appropriate protection strategies and countermeasures to ensure the safe operation of the system.

### 3.4. Risk Assessment

Our method will add a risk assessment component after the STPA process to comply with international standards. During the risk assessment phase, we provide a uniform process for complying with the HARA and TARA risk assessment processes. It is important to note that, for the determination of the risk level, the results of the risk assessment may differ from one team to another. This is because they may have different understanding of the same loss scenario. Therefore, the unified risk matrix in our method is only used as a reference, and different teams can modify the unified risk matrix according to the actual situation. For teams that do not need to quantify risk, the hazard/threat analysis part of our method can still give a more systematic and in-depth way to understand the safety and risk of a system.

Section 3.4.1 and Section 3.4.2 briefly introduce the HARA process of ISO 26262 and the TARA process of ISO/SAE 21434, and Section 3.4.3 introduces our unified risk assessment process.

#### 3.4.1. Hara Process

The HARA process consists of scenario analysis, hazard identification, and ASIL determination. Scenario analysis identifies operational situations and modes of operation that can lead to the occurrence of a hazardous event. The purpose of hazard identification is to identify hazardous events and their consequences. Finally, the ASIL for each hazardous event was determined using the Severity (from S0 to S3, representing low to high severity of potential harm of hazardous events), Exposure (from E0 to E4, representing low to high probability of exposure to operational situations), and Controllability (from C0 to C3, representing high to low probability of avoiding a specific injury) described in ISO 26262. There are four ASILs defined, where ASIL A is the lowest safety integrity level and ASIL D is the highest one. In addition to these four ASILs there is a Quality Management (QM) level, which indicates that there are no safety requirements to be complied with.

#### 3.4.2. Tara Process

**Figure 5:**
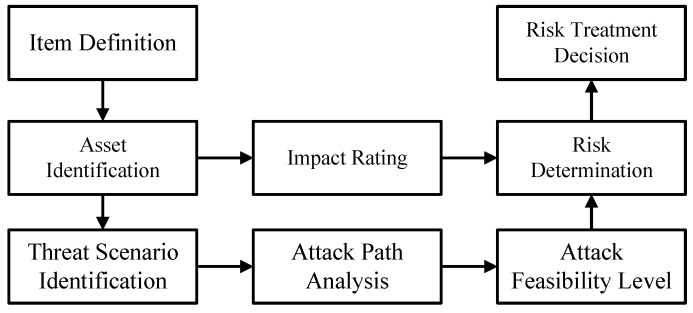
Flow diagram of TARA.

The purpose of TARA is to identify potential threats and security vulnerabilities early in the vehicle product development process. The risk level of threats is determined by the attack feasibility and impact rating, which leads to the corresponding cybersecurity goals and forms the cybersecurity requirements. There are eight main steps in the TARA process, including item definition, asset identification, threat scenario identification, impact rating, attack path analysis, attack feasibility level, risk determination, and risk treatment decision, as shown in Figure 5.

According to ISO/SAE 21434, the impact rating of the identified loss scenarios needs to be assessed in terms of Safety (S), Financial (F), Operational (O), and Privacy (P).

We use an attack potential-based approach to evaluate the attack feasibility, as shown in Table 3 and Table 4. For a malicious attack, the values of the factors in Table 3 are summed to obtain the leftmost value in Table 4, which can then be mapped to attack feasibility. The attack potential describes the difficulty of launching an effective attack. Higher attack feasibility corresponds to lower attack potential because most possible attackers have the necessary attack potential. Conversely, lower attack feasibility corresponds to higher attack potential because the number of attackers with the necessary attack potential is relatively small at this point. Factors to consider when evaluating attack potential are as follows:

1. Elapsed time (ET): Indicates the time it takes for an attacker to recognize and exploit a vulnerability to successfully execute an attack.
2. Knowledge of system (K): Indicates the difficulty of obtaining information about the target system.
3. Expertise (Ex): Attacker’s expertise and experience.
4. Window of opportunity (W): Indicates whether a special window of opportunity is required to perform the attack, including the type of access control (such as remote, physical) and the duration of access (such as restricted, unrestricted).
5. Equipment (Eq): Indicates the hardware, software, or other related equipment required by the attacker to realize the attack.

Finally, we need to calculate the risk level by referring to the risk matrix based on the impact rating and attack feasibility. An example of a risk matrix is provided in ISO/SAE 21434, where the risk values are categorized into five classes from 1 to 5, indicating the risk level from low to high.

#### 3.4.3. Unified Risk Assessment

**Figure 6:**
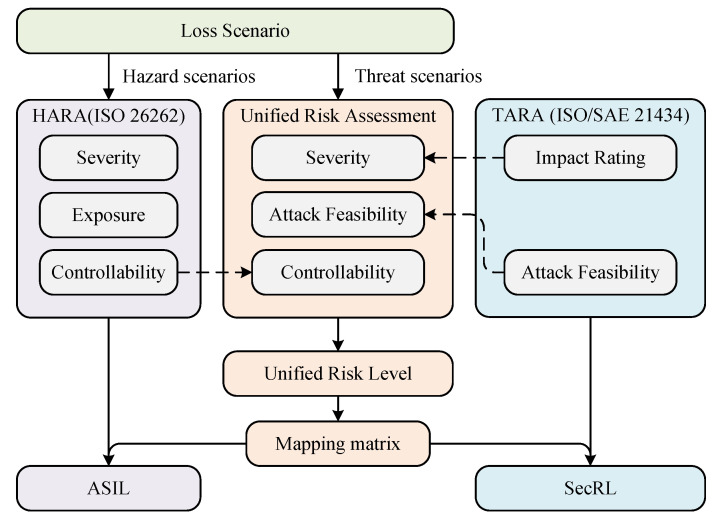
Unified risk assessment.

The unified risk assessment process is shown in Figure 6. For the loss scenarios after performing the hazard/threat analysis, we consider the following processes: if the loss scenario is a hazard scenario related only to accidental failures, then we perform only the HARA process, and if the loss scenario is a threat scenario related to malicious attacks, then we perform the unified risk assessment process.

In the unified risk assessment process, we use three parameters to determine the unified risk level:

1. Controllability: We use controllability to measure the likelihood that the driver or others at potential risk will avoid harm. The incorporation of controllability into the TARA process was demonstrated by Bolovinou et al. [52]. In order to maximize compliance with international standards and reduce the complexity of the assessment, we follow the definition of controllability in HARA (ISO 26262), as shown in Table 5. By taking into account the influence of human factors on the risk level, it will result in a more comprehensive risk assessment.
2. Severity: As shown in Figure 6, the severity parameter in the unified risk assessment are derived from TARA’s impact rating. We evaluate the severity of loss scenarios through four factors: Safety (S), Financial (F), Operational (O), and Privacy (P), which are categorized as shown in Table 6. In these four factors, the Safety factor is the same as the severity parameter of ISO 26262 (see Section 3.4.1). To simplify the calculation of severity, we use the maximum value in the severity vector ($\vec{S}$ = ($S_{S}$, $S_{F}$, $S_{O}$, $S_{P}$)) to represent the overall severity of the loss scenario. For example, if a loss scenario has a severity vector $\vec{S}$ = [2, 2, 1, 1], then the severity of this loss scenario is 2.
3. Attack Feasibility: The attack feasibility in the unified risk assessment process is derived from the attack potential-based approach recommended by ISO/SAE 21434, which we have described in detail in Section 3.4.2. We first calculate the attack potential of the loss scenario based on Table 3, and then map the attack potential to the attack feasibility level based on Table 4.

Finally, we mapped the Controllability, Severity, and Attack Feasibility of the loss scenarios to each risk level, as shown in Table 7. Risk levels range from 0 to 7+, where a rating of 7+ signifies that the risk exceeds the normal acceptable level. The same risk mapping table is used for both safety-related and security-related scenarios. The use of a unified risk matrix ensures that uniform standards are applied to safety and security risk assessments. At the same time, this increases the interaction between safety and security and simplifies the entire process. After calculating the unified risk level, we mapped it to the ASIL of ISO 26262 and the SecRL of ISO/SAE 21434, respectively, through the correspondence in Table 8.

## 4. Case Study

In this section, we will demonstrate the detailed flow of the proposed method using the example of an AEB system for ICV.

### 4.1. System Description

**Figure 7:**
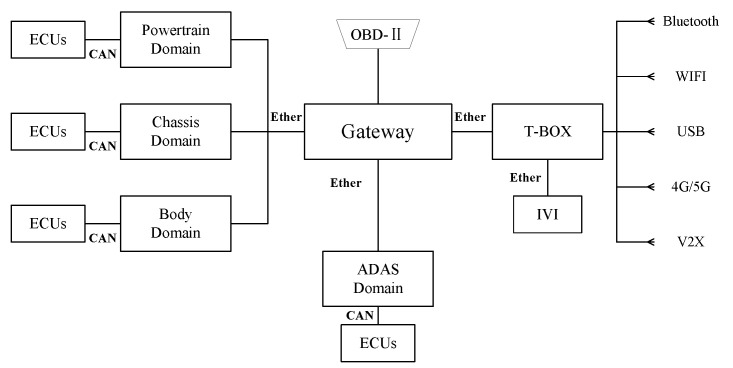
Domain-based vehicle electrical and electronic architecture.

With the innovation in Electronic and Electrical Architecture (EEA), vehicles are transitioning from traditional mechanical systems to autonomous driving systems. A large number of ECUs and sensors are integrated into vehicle systems. Figure 7 illustrates a domain-based EEA that divides the vehicle into different domains according to their functions, including powertrain domain, chassis domain, body domain, ADAS domain, and intelligent cockpit domain. Data transfer between each domain is performed via Ethernet, and within each domain, components communicate internally with each other using different bus systems.

### 4.2. Hazard/Threat Analysis

#### 4.2.1. Define Purpose of the Analysis

##### Identifying Losses

For ICVs, the human driver and the ADAS system share responsibility for controlling vehicle behavior. Due to the existence of multiple communication technologies, there are multiple interfaces for data exchange between the vehicle and the outside world. Cyberattacks launched through these exposed interfaces can not only lead to data leakage, but even cause harm to the vehicle and the people involved. Thus, the losses that should be avoided are shown in Table 9.

##### Identifying System-Level Hazards/Threats and Constraints

System-level hazards/threats are system states that can lead to loss under specific environmental conditions. Some of the relevant system-level hazards/threats are listed in Table 10. In general, a hazard/threat may lead to one or more losses, and for each hazard/threat the possible loss should be specified. Once system-level hazards/threats have been identified, it is straightforward to identify the system-level constraints that must be enforced.

#### 4.2.2. Modeling the Control Structure

**Figure 8:**
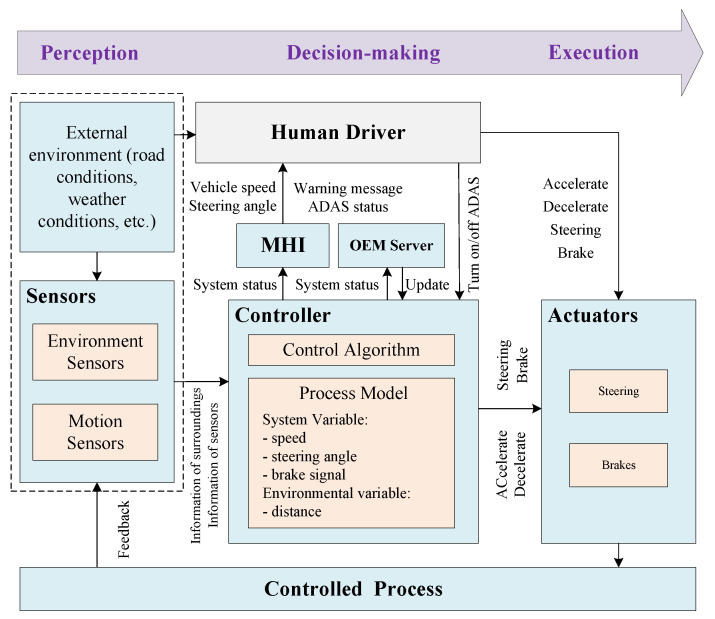
ICV control structure.

As shown in Figure 8, an ICV consists of three main functions: perception, decision-making, and execution. The perception function is achieved through various sensor components, such as LiDAR, cameras, etc. These sensors are primarily used to perceive the surrounding environment, including roads, vehicles, pedestrians, etc. We mapped the perception function to the sensors in the control structure, which are used to collect information about the vehicle’s motion state and the surrounding environmental conditions to provide accurate data support for the controller’s decision-making. The decision-making function corresponds to the controller in the control structure. It is mainly used to process data from sensors based on control algorithms and process models and then sends control commands to actuators. The process model mainly includes system variables (including the vehicle’s current speed, steering angle, brake signals, etc.) as well as environmental variables (mainly the distance between the vehicle and obstacles). The Controller’s firmware can be updated via the Internet by downloading the software package from the OEM server. The execution function corresponds to the actuator in the control structure, which mainly controls the vehicle’s acceleration, braking, steering, and other forms of action.

While ICVs are capable of making correct decisions based on environmental conditions and vehicle driving information in certain situations, in some complex driving scenarios, such as rainy weather or unclear road markings, human drivers are still required to continuously monitor the driving environment, as well as promptly detect and respond to unexpected situations. Therefore, we include the human driver in the control structure diagram and describe the interaction with the system. The human driver can observe the status of the vehicle as well as the ADAS through the Human–Machine Interface (HMI), while the driver can also observe the external environment and make the final decision.

**Figure 9:**
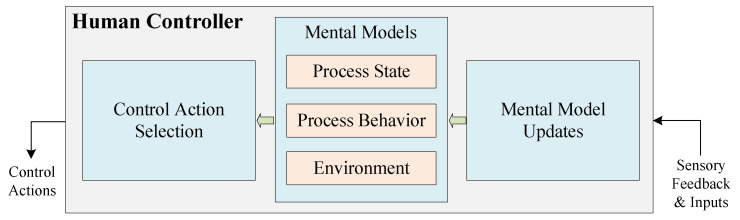
Human driver model.

When analyzing loss scenarios of UCAs from a human driver, human mental models need to be applied. For the human driver, a new model and method are proposed by France [53] to support the creation of robust causal scenarios, which are shown in Figure 9. The human driver model is divided into three parts: Control Action Selection, Mental Models, and Mental Model Updates. The Control Behavior Selection part aims to explain the reasons why a human driver chooses a specific control action. The Mental Models include the driver’s understanding of the controlled process (both its state and behaviors) and the driver’s understanding of the environment. If Mental Models are partial representations of the controlled process and the environment, Mental Model Updates are the processes by which elements of the driver’s surroundings are selectively incorporated into those representations.

##### Mapping Control Structures to Physical Component Diagrams

**Figure 10:**
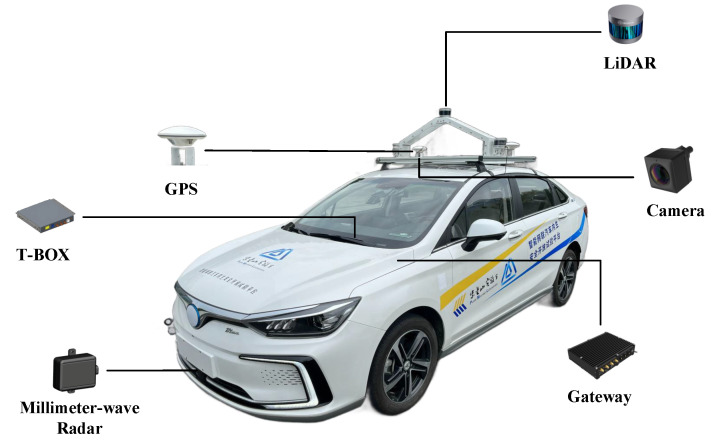
Experimental vehicle platform.

Once the control structure diagram of the system was constructed, we mapped it to a physical component diagram based on the physical structure of the system. Vehicle systems are categorized into several domains according to their functions. Among them, ADAS is used to enable environmental perception and decision-making. Chassis domain is used to enable braking and steering control. Infotainment domain includes dashboard and infotainment system. The in-vehicle network is connected to the external network via T-box for remote control, remote updates, etc. According to our experimental vehicle platform, the components related to AEB are shown in Figure 10.

**Figure 11:**
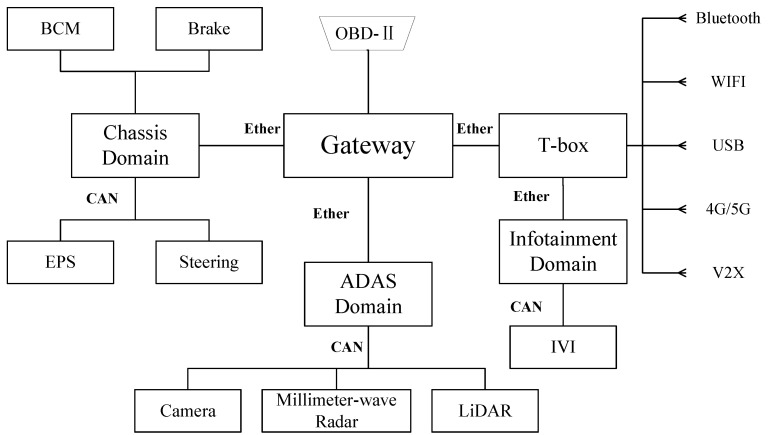
AEB system Architecture.

Furthermore, Figure 11 shows a diagram of a system architecture consisting of components and networks associated with the realization of the AEB system, which can be considered as a subsystem of ADAS. The AEB system uses sensors to measure the distance to other vehicles or obstacles. The vehicle will raise an alarm when the measured distance is less than the warning distance. When the measured distance is less than the safe distance, the AEB system will brake the vehicle. The sensors involved include LiDAR and camera. The functions provided by AEB are specified as follows:

1. Collision warning: Continuous monitoring of the distance to the vehicle in front through sensors, sending warning signals to IVI according to the distance to the vehicle in front of it.
2. Collision mitigation: ADAS sends a command to the Brake Control Module (BCM) when the sensors detect that the vehicle in front is too close. If the driver reacts urgently but braking force is insufficient, additional braking force is provided.
3. Emergency braking: When the sensors detect that the vehicle in front is too close, if the driver does not respond to the warning, the ADAS will send a deceleration command to the BCM and send a lock command to the Electric Power Steering (EPS).

**Figure 12:**
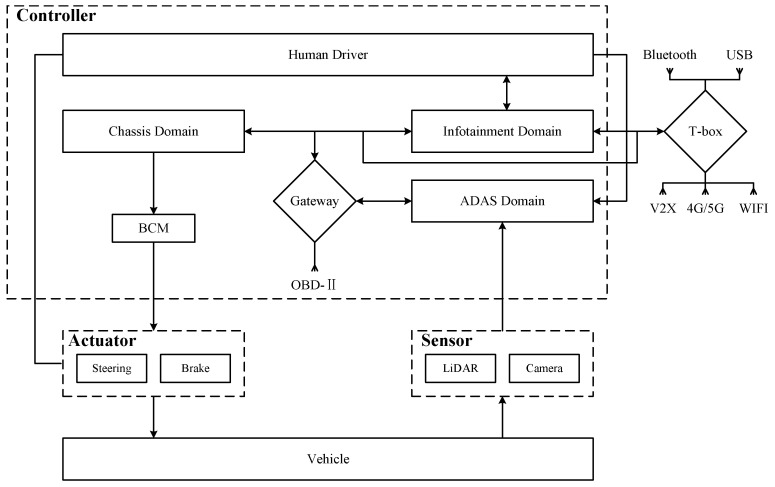
Physical component diagram of AEB.

Based on the generic physical component diagram and AEB system architecture, the control structure diagram is mapped to the physical component diagram shown in Figure 12. The human driver, as part of the controller, can control whether the ADAS system is turned on or not as well as react to alarms displayed by the IVI. The ADAS domain control unit is responsible for receiving environmental information collected by LiDAR and the camera. Based on the collected data, it uses specific algorithms to analyze and assess the vehicle’s driving conditions, perceiving potential collision risks. Finally, it sends decision information to both the chassis domain control unit and the infotainment domain control unit to control braking, steering lock, and collision warnings.

#### 4.2.3. Identifying Unsafe/Unsecure Control Actions

Based on the system control structure diagram, the UCAs are analyzed. The main control actions provided by the controller are acceleration, deceleration, brake, steering, etc. Based on the guide words, it is possible to list the ways in which these control actions may be unsafe/unsecure. Table 11 lists the specific descriptions of unsafe control actions related to brakes.

The unsecure control actions are mainly related to vehicle data transfer processes, such as remote updates of ECU firmware. Table 12 lists the unsecure control actions related to the OEM Server.

The loss scenarios that lead to unsecure control actions are usually included in the loss scenarios for unsafe control actions. In other words, safety can often be compromised as a result of security problems. For example, an attacker could install malware on a vehicle’s ECU via an over-the-air upgrade (security), which could lead to a serious accident (safety). Therefore, in the loss scenario identification phase, we focus on unsafe control actions because they also include loss scenarios for unsecure control actions.

The control structure also allows us to identify unsafe control actions related to the human drivers. The human driver can monitor the status of the vehicle’s ADAS system through the HMI. At the same time, the human driver can make a series of control actions according to the actual road environment, and there are unsafe situations for these control actions. The descriptions of unsafe control actions related to braking for the human driver are presented in Table 13.

#### 4.2.4. Identifying Loss Scenarios

##### Identifying Loss Scenarios Related to Human Driver

According to Figure 9, there are three questions to consider when identifying loss scenarios for unsafe control actions associated with human drivers: (1) How did the operator choose which control action to perform? (2) What does the operator know or believe about the system? and (3) How did the operator come to have their current knowledge or beliefs? These three questions correspond to the three parts of the human driver model. Table 14 shows the process of identifying a human driver-related loss scenario.

For Q1, we are considering factors related to Control Action Selection. We need to analyze why the driver did not apply the brakes. One possible reason is that the driver may believe that with ADAS activated, emergency braking is not required in most situations.

For Q2, we consider the driver’s knowledge or beliefs about the state of the system. First, the mental model of Process State usually refers to beliefs regarding the current state of the system, e.g., the state the driver believes the system is in. So one possible situation is that the driver thinks the ADAS is operating normally, but in fact the ADAS is in an abnormal state. Next, we consider the mental model of Process Behavior, including what the driver thinks the system can do, what he or she can do, and how the system will react to his or her behavior. One possible factor is that the driver is aware that the AEB subsystem of the ADAS will engage braking in certain emergency situations. Finally, we consider the mental model of Environment, as these beliefs about the environment can influence the selection of control actions. One possible situation is that the driver does not notice the obstacle ahead.

For Q3, it is necessary to consider how the driver’s incorrect beliefs are formed. One possible situation is that the ADAS status displayed by the HMI is wrong due to some malfunction.

To summarize, a loss scenario related to a human driver can be described as follows: the driver did not apply the brakes when the vehicle was less than a safe distance from the obstacle (vehicle) in front of it. This is because the driver follows the rule that “when the ADAS is on, he does not need to apply the emergency brake himself in most cases”. The driver incorrectly believes that the ADAS system is operating normally by observing the information from the HMI and fails to notice an obstacle just a short distance ahead. In fact, due to a system failure, the HMI display was incorrect.

##### Modeling Loss Scenario Trees

Based on the physical component diagram of the vehicle, we first define all the components that could be subject to security threats, including LiDAR, cameras, T-BOX, ADAS domain control units, chassis domain control units, and infotainment domain control units, etc.

For LIDAR, we consider two types of security threats, Spoofing and Denial of Service (DoS), based on the STRIDE threat model. In addition, it also has functional failure possibilities, which should also be reflected in the loss scenario tree. After identifying possible threat scenarios for each component, these scenarios are then connected to possible attack paths. An attack path describes the steps taken by an attacker to launch an attack. Analysts can determine the mitigations needed to protect a system based on a series of attacker behaviors.

**Figure 13:**
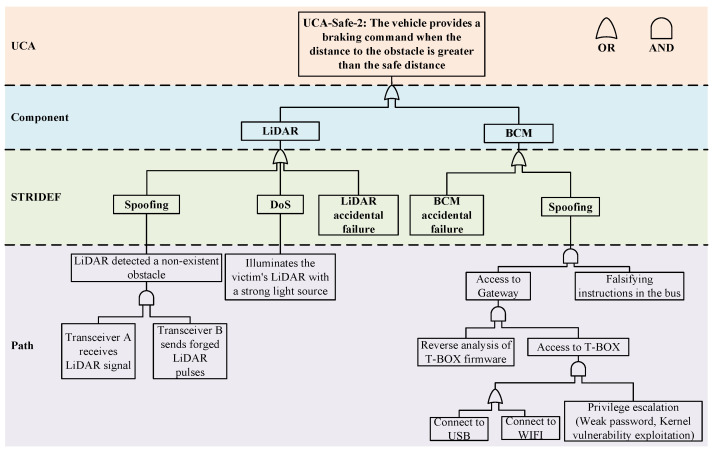
Loss scenario tree associated with UCA-Safe-2.

Figure 13 shows the specific loss scenario tree with "UCA-Safe-2: The vehicle provides a braking command when the distance to the obstacle is greater than the safe distance" as the root. We describe in detail the Spoofing attack and DoS attack against LiDAR and the Spoofing attack against BCM.

LiDAR is a ranging sensor capable of measuring the distance and shape of surrounding objects by sending laser pulses and receiving their reflections [54]. In an ICV, the main role of LiDAR is to provide high-precision environment sensing capability to help vehicles achieve safe autonomous driving. The Spoofing attack against LiDAR is described in the Step layer, which is mainly realized by two transceivers. Transceiver A is a photosensitive detector sensitive to the wavelength of the laser emitted by the LiDAR. Its output is a voltage signal, which corresponds to the intensity of the laser pulse emitted by the LiDAR. The output of transceiver A is sent to transceiver B, which ultimately sends a laser pulse to the LiDAR for the attack. During a Spoofing attack, LiDAR anomalously detects non-existent obstacles, which in turn can affect subsequent path planning and lead to a root event.

LiDAR can be thought of as a transducer that primarily converts one type of input into another. While the transition curve of LiDAR is linear for the most part, a certain degree of non-linearity is unavoidable when the input light intensity is high. Based on the intensity of the input, it can be categorized into silent region, linear region, and saturation region. The silent region represents input intensity that does not reach the sensor’s activation threshold. The linear region is the intensity range within which the sensor operates normally, and the saturation region represents input intensities that are too high, at which point the sensor cannot effectively respond to changes in input. Thus, a DoS attack against a LiDAR can be described as an attacker using specialized equipment to emit light at the same wavelength as that used by the target LiDAR, which is strong enough to saturate the target LiDAR.

The function of the BCM is to control and regulate the vehicle’s braking system to ensure the braking performance and safety of the vehicle. It receives information from other components of the vehicle, such as the position of the brake pedal, vehicle speed, tire speed, etc., in order to dynamically adjust and control the braking system. At the same time, ADAS can also send commands to the chassis DCU via the central gateway, which in turn transmits braking commands to the BCM. T-BOX, as the most important communication terminal in the vehicle, is mainly responsible for communicating with the remote cloud in the vehicle. The attacker uses specialized equipment to connect to WIFI, USB, and other interfaces, and then gains root access to the T-BOX through methods such as brute-force attacks on weak passwords and exploitation of kernel vulnerabilities. By analyzing the internal firmware code, the attacker cracks the content of message sessions, enabling tampering with data transmitted through the protocol. This allows for the modification of user commands or the transmission of forged commands onto the bus, enabling unauthorized remote control of the vehicle.

#### 4.2.5. Refining Safety/Security Constraints

In Table 10, we initially obtain the system-level constraints corresponding to system-level hazards/threats. With the loss scenario tree, we can further refine these constraints based on fault propagation paths and attack paths, and the results are shown in Table 15.

As shown in Table 16, we marked the safety constraints and security constraints that conflict with each other. We can find constraints related to encryption and authentication that may introduce additional computational and communication overheads, which may increase the response time of the system, thus reducing its responsiveness to emergency situations. Therefore, system design needs to find a balance between safety and security to ensure that security is improved without sacrificing safety.

### 4.3. Risk Assessment

The purpose of the risk assessment is to calculate the risk level based on controllability, severity, and attack feasibility. In the loss scenario tree, we list the possible attack paths as well as failure modes for components related to UCA-Safe-2. For each attack path, we use the attack potential to rate the attack feasibility, which mainly includes five aspects: Elapsed time, Knowledge of system, Expertise, Window of opportunity, and Equipment, as shown in Table 17.

For example, for a Spoofing attack against BCM, the time required for the attack includes the time to identify the T-BOX kernel vulnerability and the time to exploit the vulnerability, which may be less than a week. Since information about the target T-BOX cannot be obtained through public channels, we set Knowledge to sensitive. Since firmware analysis for the T-BOX and forging bus commands both require the attacker to have in-depth knowledge of algorithms, protocols, hardware, and cryptography in the underlying domain, we set Expertise to expert. The first step in the attack path requires the use of a device to connect to the vehicle’s WIFI or Bluetooth, which requires the attacker to be in close proximity to the vehicle, so its Window of opportunity is set to easy. The Equipment used to accomplish the BCM Spoofing attack is relatively easy for an attacker to obtain, requiring only a laptop. Based on Table 4 and the scores of all dimensions of attack potential, we obtain the attack feasibility level of BCM Spoofing attack.

For UCA-Safe-2, the Severity is considered and evaluated on four main dimensions: Safety, Financial, Operational, and Privacy. UCA-Safe-2 describes a situation in which the vehicle provides a braking command when the distance to an obstacle is greater than the safe distance. Since STPA is a worst-case analysis method [15], we consider the worst-case scenario: if a vehicle is on a highway and is traveling at a high speed, unexpected braking may not only cause the vehicle to deviate from its original route, but may also result in a rear-end collision. According to the situation described in UCA-Safe-2, this could have a major impact on people’s safety, financial damage, and the operational function of the vehicle; therefore, we obtain the severity vector S = [2, 2, 2, 0]. To simplify the analysis, we take the maximum value in the vector to represent the impact rating [27]. In addition, since the hazard occurs when the vehicle is traveling at high speeds and it is difficult for the driver to control the hazardous event, it has a Controllability rating of 3. Combining the attack feasibility of each attack path, we finally determine the unified risk level as 6 through the risk matrix in Table 7. The unified risk level is mapped to ISO26262 and ISO/SAE 21434 through the risk level mapping relationship in Table 8, and the results are shown in Table 18. Once the risk level is determined, the final step is to apply countermeasures to reduce the risk value. We then repeat the entire analysis process until the risk reaches an acceptable level.

### 4.4. Methods Comparison Analysis

According to the comparative attributes proposed in [51], we compare the differences between the method proposed in this work and several methods applicable to the vehicle domain, as shown in Table 19.

FMEA is a recommended method in ISO 26262, which identifies potential causes by listing the failure modes of a sub-function in the AEB system. Attack tree is an analysis method recommended by ISO/SAE 21434. It uses logic gates to model attack paths. ${\mathrm{US}}^{2}$ combines a security analysis to ASIL, which can evaluate threats and failures in parallel. STPA with a Six-Step Model integrates STPA into the concept phase of ISO 26262 while using a six-step model to maintain consistency between safety and security processes and artifacts. Our method is based on the improved STPA, which initially establishes the system control structure and further refines it into a physical component diagram. We focus on the control actions between system components in the physical component diagram and discuss the ways in which they are unsafe/unsecure. During the analysis, the driver is regarded as a part of the controller. This is consistent with real driving scenarios and can reflect the driver’s influence on the risk level. For the identified causal scenarios, we model them using a loss scenario tree, which facilitates analysts in adding mitigation measures at appropriate points. Our approach utilizes the STRIDE model for threat modeling while also taking into account fault scenarios. Compared to other methods, it can identify a more extensive range of causal scenarios. Our approach also uses a unified risk metric matrix that allows risk values to be mapped to the ASIL of ISO 26262 and the SecRL of ISO/SAE 21434.

## 5. Conclusions and Future Work

The rapid development of modern vehicles toward intelligence and connectivity has also brought new risks to vehicle safety. We proposed an S&S co-analysis method to address accidental failures and cybersecurity threats in vehicles. Our method is based on STPA, which identifies loss scenarios by considering unsafe/unsecure control actions. To compensate for the abstraction of the control structure diagram in STPA, we improved the physical component diagram of STPA-SafeSec by adding interface elements. The improved physical component diagram can describe a wide range of interfaces between ICVs and the outside world, thus allowing for the identification of a wider and more detailed range of loss scenarios. In addition, we proposed a loss scenario tree consisting of four layers to deeply analyze loss scenarios including accidental failures and malicious attacks. With the loss scenario tree, we describe in detail the fault propagation paths and attack paths that lead to UCA. Based on the identified loss scenarios, we can further refine the safety/security constraints, which correspond to the safety/security requirements in the international standards for vehicles. For the obtained safety/security constraints, we can analyze the conflicting relationship between them to select balanced mitigation measures. In order to comply with the concept phase of ISO 26262 and ISO/SAE 21434, we have added a risk assessment process. We use a unified risk matrix to obtain the risk level of loss scenarios and map the risk level to ASIL and SecRL, respectively. Finally, we conducted a case study of the AEB system. By analyzing the established control structure diagram and physical component diagram, we obtained the loss scenario trees and the risk level of the scenarios. The results indicate that our proposed method can meet the requirements of the concept phase of the vehicle international standards and provide more comprehensive and effective recommendations in the early stages of vehicle development.

In future work, we plan to explore ways to more effectively quantify the risk level of loss scenarios, which will help prioritize different risks. For the risk mitigation measures developed, quantifying their impact on the level of risk can help analysts determine which measures should be implemented primarily to minimize potential risks, and thus allocate resources and manage risks effectively.

## Figures and Tables

**Table 1:** Comparison of analysis methods.

Method	Type	Analysis Results (Qualitative/Quantitative)	Causal Factors for Loss Scenarios	Level of Analysis (Abstract/Specific)	Comply with Standard (Y/N)
STPAFT [26]	Safety	Qualitative and quantitative	Component failure (including unsafe and unexpected interactions between system components)	Abstract and Specific	N
VeRA [27]	Security	Qualitative and quantitative	Malicious attacks	Specific	Y
STPA-Sec [16]	Security	Qualitative	Component failure (including unsecure and unexpected interactions between system components)	Abstract	N
${\mathrm{US}}^{2}$ [28]	S&S	Qualitative and quantitative	Component failure and malicious attacks	Specific	Y
STPA with Six-Step Model [29]	S&S	Qualitative	Component failure and malicious attacks (including unsafe/unsecure and unexpected interactions between system components)	Specific	Y
STPA-SafeSec [14]	S&S	Qualitative	Component failure and malicious attacks (including unsafe/unsecure and unexpected interactions between system components)	Abstract and Specific	N
Unified safety and cybersecurity analysis method [30]	S&S	Qualitative and quantitative	Component failure and malicious attacks (including unsafe/unsecure and unexpected interactions between system components)	Abstract	Y

**Table 2:** Foundations of ISO 26262, ISO/SAE 21434 and our method.

	ISO 26262 (HARA)	ISO/SAE 21434 (TARA)	Our Method
Phase	Concept phase	Concept phase	Apply at any phase of system design, including the concept phase
Characteristics	Develop clear safety goals and corresponding safety requirements Continuous refinement of safety requirements during the vehicle development cycle	Develop clear security goals and corresponding security requirements Continuous refinement of security requirements during the vehicle development cycle	Continuously refine safety/security constraints in subsequent steps after determining them
Risk assessment	Determine ASIL based on severity, exposure, and controllability	Determine security level based on impact rating and attack feasibility	Risks are assessed and mapped to ASIL level and security level using the same risk matrix
Results	Categorize hazard events according to ASIL levels while identifying safety constraints and safety requirements to avoid unreasonable risks	Identify potential threats to vehicles and their risk levels to clarify cybersecurity objectives and generate cybersecurity requirements	Identify system-related hazards or threats, determine their causal scenarios, and develop safety and security constraints to eliminate, mitigate, or control them

**Table 3:** Factors for calculating attack potential (from ISO/SAE 21434).

Factor	Level	Value
Elapsed time	≤1 day	0
	≤1 week	1
	≤1 month	4
	≤3 months	10
	≤6 months	17
	>6 months	19
	not practical	*∞*
Expertise	Layman	0
	Proficient	3
	Expert	6
	Multiple experts	8
Knowledge of system	Public	0
	Restricted	3
	Sensitive	7
	Critical	11
Window of opportunity	Unnecessary/unlimited	0
	Easy	1
	Moderate	4
	Difficult	10
	None	*∞*
Equipment	Standard	0
	Specialized	4
	Bespoke	7
	Multiple bespoke	9

**Table 4:** Attack feasibility (defined in ISO/SAE 21434).

Values	Attack Potential Required to Identify and Exploit Attack Path	Attack Feasibility	
0–9	Basic	5	High
10–13	Enhanced-Basic	4	High
14–19	Moderate	3	Medium
20–24	High	2	Low
≥25	Beyond High	1	Very low

**Table 5:** Classes of controllability (defined in ISO 26262).

C0	C1	C2	C3
Controllable in general	Simply controllable	Normally controllable	Difficult to control or uncontrollable

**Table 6:** Classes of severity (defined in ISO/SAE 21434).

Safety ($S_{S}$)	Financial ($S_{F}$)	Operational ($S_{O}$)	Privacy ($S_{P}$)
0	0	0	0
1	1	1	1
2	2	2	2
3	3	3	3

**Table 7:** Unified risk matrix.

Controllability	Severity	Attack Feasibility				
		AF = 1	AF = 2	AF = 3	AF = 4	AF = 5
C = 0	S = 0	0	0	1	2	3
	S = 1	0	1	2	3	4
	S = 2	1	2	3	4	5
	S = 3	2	3	4	5	6
C = 1	S = 0	0	1	2	3	4
	S = 1	1	2	3	4	5
	S = 2	2	3	4	5	6
	S = 3	3	4	5	6	7
C = 2	S = 0	1	2	3	4	5
	S = 1	2	3	4	5	6
	S = 2	3	4	5	6	7
	S = 3	4	5	6	7	7+
C = 3	S = 0	2	3	4	5	6
	S = 1	3	4	5	6	7
	S = 2	4	5	6	7	7+
	S = 3	5	6	7	7+	7+

**Table 8:** Mapping matrix for unified risk level.

Unified Risk Level	ASIL	Security Risk Level
1	QM	1
2	A	2
3	A	2
4	B	3
5	B	3
6	C	4
7	D	5
7+	Risk deemed beyond normally acceptable levels	

**Table 9:** Identified losses.

Loss	Description
L-1	Damage to the safety of persons
L-2	Vehicle damage
L-3	Sensitive data leakage

**Table 10:** System-level hazards/threats.

Hazard	Description	Constrains
H-1	Vehicle did not maintain a safe distance from surrounding vehicles or obstacles during driving [L-1, L-2]	SC-1: Vehicle must maintain a safe distance from surrounding vehicles or obstacles during the driving process
H-2	Damage to the physical integrity of the vehicle [L-1, L-2]	SC-2: Vehicle must maintain physical integrity
H-3	Vehicle off the planned route [L-1, L-2]	SC-3: Vehicle must keep the planned route
H-4	Vehicle unable to perform its functions [L-1, L-2]	SC-4: Vehicle must perform its functions properly
T-1	Vehicle is subject to unauthorized remote access [L-1, L-2, L-3]	SC-5: Remote access to vehicles must be authorized

**Table 11:** Unsafe control actions related to brake (Controller).

Control Action	Unsafe Control Action	
	*Not Providing*	*Providing*
**Brake**	UCA-Safe-1: The vehicle did not provide braking command when the distance to the obstacle is less than the safe distance [H-1, H-2]	UCA-Safe-2: The vehicle provides braking commands when the distance to the obstacle is greater than the safe distance [H-1, H-2]
	*Provided but wrong timing*	*Provided but incorrect duration*
	UCA-Safe-3: The vehicle provides braking command, but too early [H-1, H-2] UCA-Safe-4: The vehicle provides braking command, but too late [H-1, H-2]	UCA-Safe-5: The vehicle provides braking command, but the duration is too long [H-1, H-2] UCA-Safe-6: The vehicle provides braking command, but stops too early [H-1, H-2]

**Table 12:** Unsecure control actions related to Update (OEM Server).

Control Action	Unsecure Control Action	
	*Not Providing*	*Providing*
**Update**	UCA-Sec-1: OEM Server does not update firmware for vulnerable ECUs [H-4, T-1]	UCA-Sec-2: OEM Server updates ECU firmware, leading to new vulnerability [H-4, T-1]
	*Provided but wrong timing*	*Provided but incorrect duration*
	-	UCA-Sec-3: OEM Server firmware update process stopped too soon [H-4, T-1]

**Table 13:** Unsafe control actions related to brake (human driver).

Control Action	Unsafe Control Action	
	*Not Providing*	***Providing***
**Brake**	UCA-Safe-7: The human driver does not provide braking action when the vehicle is less than a safe distance from an obstacle [H-1, H-2]	UCA-Safe-8: The human driver provides braking action, but not enough [H-1, H-2]
	*Provided but wrong timing*	*Provided but incorrect duration*
	UCA-Safe-9: The human driver provides braking action too late, when the distance to the obstacle is less than the safe distance [H-1, H-2]	UCA-Safe-10: The human driver stops the braking action sooner than necessary when the current speed is still higher than the threshold value [H-1, H-2]

**Table 14:** The process of identifying loss scenarios related to human driver.

UCA	Q1	Q2	Q3
UCA-Safe-7: The human driver does not provide braking action when the vehicle is less than a safe distance from an obstacle [H-1, H-2]	1. The human driver believes that, with ADAS activated, he or she does not need to perform emergency braking in most situations.	2a. The human driver incorrectly believes that the ADAS system is operating normally and does not require manual braking.	3. The HMI displays that the ADAS is operating normally, but in reality, the HMI display is incorrect or experiencing delayed status updates.
		2b. The human driver knows that the AEB subsystem of ADAS will provide an emergency braking command.	
		2c. The human driver fails to notice an obstacle in front of the vehicle.	

**Table 15:** Refinement of constraints.

UCA	System-Level Constrains	Safety/Security Constrains
UCA-Safe-2: The vehicle provides a braking command when the distance to the obstacle is greater than the safe distance [H-1, H-2]	SC-1: Vehicle must maintain a safe distance from surrounding vehicles or obstacles during the driving process [H-1]	SafeC-1.1: The vehicle’s sensors (e.g., LiDAR, camera, etc.) need to be able to accurately detect and measure the position and speed of surrounding vehicles and obstacles
		SafeC-1.2: Redundant design of sensors
		SafeC-1.3: Uses high-performance information fusion
		SafeC-1.4: Reduces the transmission delay of data between nodes
		SafeC-1.5: Reduces the computational latency of information received by the T-BOX
		SafeC-1.6: Reduced latency in V2V communications
		SecC-1.1: Ensure the integrity of sensor (e.g., LiDAR, camera, etc.) data to prevent data tampering or falsification
		SecC-1.2: Encryption and authentication of communications between vehicles to ensure their protection from malicious attacks
		SecC-1.3: Equipped with intrusion detection systems to defend and protect against malicious attacks
		SecC-1.4: Force authentication on each node (ECU)
	SC-2: Vehicle must maintain physical integrity [H-2]	SafeC-2.1: Regular inspection and maintenance of vehicles to ensure that they are not structurally damaged or corroded
		SafeC-2.2: Restrictions on the operation of vehicles in specific situations

**Table 16:** Conflicts between safety and security constraints. ✓ indicates a conflict between safety and security constraints.

	SafeC-1.4	SafeC-1.5	SafeC-1.6
SecC-1.1	✓		
SecC-1.2		✓	✓
SecC-1.3	✓	✓	
SecC-1.4	✓		

**Table 17:** Attack feasibility of attack paths against BCM and LiDAR.

Threat Description	Attack Path	Attack Feasibility					
		ET	K	Ex	W	Eq	**Attack** **Feasibility** **Rating**
Spoofing attack of BCM	1. Connect to the vehicle’s WIFI or Bluetooth via cell phone	1	7	6	1	0	3
	2. Gain root access to T-BOX by exploiting weak password cracking or kernel vulnerabilities						
	3. Reverse Analyzing T-BOX Firmware						
	4. The attacker forges T-BOX commands and forwards malicious instructions to other DCUs through the gateway						
	5. DCU sends malicious commands to the BCM						
DoS attack of BCM	1. The attacker gains access to the in-vehicle network by connecting to the IVI through professional tools	1	7	6	1	0	3
	2. Transmitting malicious control signals through the compromised IVI						
	3. The attacker compromises the central gateway						
	4. The gateway forwards malicious signals to DCUs						
	5. The attacker floods the bus connecting the DCU to the BCM with a large number of malicious signals						
Spoofing attack of LiDAR	1. Attacker receives optical signals using transceiver A	1	3	2	4	4	3
	2. Transceiver B receives the voltage signal from A and sends an optical signal to the LiDAR						
DoS attack of LiDAR	1. Attackers use oversaturation attacks against LiDAR	1	3	6	4	4	3

**Table 18:** Risk mapping of physical components.

UCA	Impact Rating	Controllability	Component	RL	ASIL	SecRL
UCA-Safe-2: The vehicle provides a braking command when the distance to the obstacle is greater than the safe distance [H-1, H-2]	[2, 2, 2, 0]	3	BCM	6	C	4
			LiDAR	6	C	4

**Table 19:** Comparison of FMEA, ${\mathrm{US}}^{2}$, Attack tree, STPA with Six-Step Model, and our method.

Attribute	FMEA	Attack Tree	${\mathrm{US}}^{2}$	STPA with Six-Step Model	Our Method
Integrates human interaction	N	N	N	Y	Y
Identify hazards and threats	Hazards	Threats	Hazards and threats	Hazards and threats	Hazards and threats
Qualitative or quantitative	Qualitative and quantitative	Qualitative and quantitative	Qualitative and quantitative	Qualitative	Qualitative and quantitative
Hazard (threat) causal factors	Component failure	Malicious attack	Component failure	Component failure, unsafe/unsecure interaction between components	Component failure, unsafe/unsecure interaction between components
Perspective of analysis	Function	Component (function)	Component	Control action	Control action
Threat model	N	N	N	N	STRIDE
Complexity	Low	Low	Low	High	High
Model of failure (attack) path	N	Attack tree	N	Failure tree and attack tree	Loss Scenario Tree (failure and attack path)

## Data Availability

Data are contained within the article.

## Abbreviations

The following abbreviations are used in this manuscript:

ICV	Intelligent Connected Vehicle
CPS	Cyber-Physical System
ECU	Electronic Control Unit
CAN	Controller Area Network
HARA	Hazard Analysis and Risk Assessment
TARA	Threat Analysis and Risk Assessment
ASIL	Automotive Safety Integrity Level
SecRL	Security Risk Level
AEB	Autonomous Emergency Braking
STPA	System-Theoretic Process Analysis
